# Suppression of NLRP3 inflammasome improves alveolar bone defect healing in diabetic rats

**DOI:** 10.1186/s13018-019-1215-9

**Published:** 2019-05-30

**Authors:** Hao Li, Xinghua Zhong, Zhiyong Chen, Wei Li

**Affiliations:** 1 Department of Prosthodontics, The Affiliated Hospital of Stomatology, Guangxi Medical University, 10 Shuangyong Road, Nanning, 530021 People’s Republic of China; 2 State Key Laboratory of Oral Diseases, West China Hospital of Stomatology, Sichuan University, 14 3rd Section S Renmin Road, Chengdu, 610041 People’s Republic of China

**Keywords:** NLRP3 inflammasome, Interleukin-1, Alveolar bone, Diabetes, RNA interference

## Abstract

**Background:**

Excessive inflammatory response under hyperglycemia can impair alveolar bone defect healing under diabetic conditions. NLRP3 (NACHT [nucleotide-binding oligomerization], LRR [leucine-rich repeat], and PYD [pyrin domain] domains-containing protein 3) inflammasome has been considered to play a crucial role in the inflammatory response, but its correlation with the impaired alveolar bone repair in diabetes still remains unclarified. The objective of the current study is to investigate the effect of NLRP3 inflammasome inhibition by a lentiviral short hairpin RNA (shRNA) targeting NLRP3 on alveolar bone defect healing in diabetic rats.

**Methods:**

Diabetes was induced in rats by high-fat diet and streptozotocin injection, and alveolar bone defects in both maxillae were created by surgery. Then, the lentiviral shRNA targeting NLRP3 was applied in the defect. Eight weeks after surgery, the alveolar bone regeneration was examined using hematoxylin and eosin (H&E) staining, and the gene expression in the bone healing site was detected using quantitative real-time reverse transcription polymerase chain reaction (qRT-PCR) analysis and western blot analysis.

**Results:**

H&E staining showed that treatment with lentiviral shRNA targeting NLRP3 could increase the bone regeneration score in the alveolar bone defect of diabetic rats. Additionally, qRT-PCR analysis and western blot analysis of the bone defect demonstrated that this shRNA inhibited the expression of NLRP3, apoptosis-associated speck-like protein containing a CARD, caspase-1, and proinflammatory cytokine interleukin-1β and increased the expression of osteogenic markers Runt-related transcription factor 2 and osteocalcin.

**Conclusions:**

Our findings suggested that inhibition of NLRP3 inflammasome could improve alveolar bone defect healing in diabetic rats. The beneficial effect may correlate with reduced proinflammatory cytokine production and increased osteogenic gene expression in hyperglycemia.

**Electronic supplementary material:**

The online version of this article (10.1186/s13018-019-1215-9) contains supplementary material, which is available to authorized users.

## Background

Currently, impaired alveolar bone healing in diabetic patients still remains a great problem in dentists’ clinical work, and it can impede the implementation of different oral disease treatments, such as tooth extraction and dental implant surgery. These patients often exhibit high blood glucose, which has detrimental effects on alveolar bone repair [1, 2]. Although the exact cellular mechanisms of this impaired healing are unclear, high levels of proinflammatory cytokines, like interleukin-1β (IL-1β), in periodontal tissues under the condition of hyperglycemia are considered to be potentially important contributors [1]. These cytokines can exacerbate inflammation and subsequently inhibit tissue formation.

NLRP3 (NACHT [nucleotide-binding oligomerization], LRR [leucine-rich repeat], and PYD [pyrin domain] domains-containing protein 3) plays a crucial role in inflammatory cytokine production and inflammation response [3]. It assembles a multi-protein complex called inflammasome, which is composed of NLRP3, apoptosis-associated speck-like protein containing a CARD (ASC), and caspase-1 [4]. Upon activation, NLRP3 recruits ASC by pyrin domain interactions and subsequently recruits procaspase-1 through CARD-CARD interactions [5, 6]. Then, procaspase-1 autocatalyzes its cleavage and results in the maturation of the precursor forms of IL-1β, leading to the secretion of other different proinflammatory cytokines [7]. Overexpression of NLRP3 inflammasome has been found in various inflammatory diseases, including delayed soft tissue wound healing and periodontitis under diabetic conditions [7, 8]. These findings suggest the inhibition of NLRP3 inflammasome may promote alveolar bone repair in diabetes.

In this work, to detect the effect of NLRP3 inflammasome suppression on the alveolar bone healing under the diabetic condition, we established a diabetic rat model of alveolar bone defect, applied a lentiviral short hairpin RNA (shRNA) targeting NLRP3 in the defect, and then examined the mRNA and protein expression of NLRP3 inflammasome, proinflammatory cytokine IL-1β, and bone formation-related factors in the bone healing site.

## Methods

### Induction of diabetes

Forty 4-week-old male Wistar Hanover rats (Laboratory Animal Center, Guangxi Medical University, China) were randomly assigned to the normal control (N), diabetes without treatment (D), diabetes with control shRNA lentivector treatment (DC), and diabetes with lentiviral NLRP3 shRNA treatment (DR) groups (10 rats in each group). All protocols were approved by the Institutional Committee for Animal Use and Care at Guangxi Medical University, and all experiments were in accordance with the Animals in Research: Reporting In Vivo Experiments (ARRIVE) guidelines [9]. Normal control rats were fed low-fat food (12 kcal% fat; Laboratory Animal Center, Guangxi Medical University, China). D, DC, and DR rats were fed high-fat food (48 kcal% fat; Laboratory Animal Center, Guangxi Medical University, China) for 2 weeks and then were intraperitoneally injected streptozotocin (STZ; Sigma-Aldrich Co., St. Louis, MO, USA) (single dose, 35 mg/kg). Blood from the rat vein tails was collected to detect the fasting blood glucose 1 week after STZ injection (7 weeks old), and fasting blood glucose over 13.89 mmol/L was considered diabetic [10].

### Establishment of alveolar bone defects and RNA interference (RNAi)

After confirmation of diabetes, all rats were fed the low-fat food until 11 weeks old. Subsequently, all of them were anesthetized by intraperitoneal injection of ketamine (7.5 mg/100 g body weight) and xylazine (1 mg/100 g body weight) and received surgery to construct bilateral maxillary alveolar bone defects (3 mm in length, 1.5 mm in width, and 1.5 mm in depth) [11]. Immediately after bone defect creation, DC and DR rats respectively received local injection of AteloGene atelocollagen gel (Koken Co., Tokyo, Japan) (a degradable material to retain the shRNA in the defect, 5 μL for each defect) containing 0.1 mol/L control shRNA lentivector (GenePharma, Shanghai, China) or 0.1 mol/L lentiviral NLPR3 shRNA (GenePharma, Shanghai, China). The lentiviral NLRP3 shRNA sequences used were as follows: forward, 5′-TGCTGATAAGAAGTTCTCTCCTGGTTGTTTTGGCCACT GACTGACAACCAGGAGAACTTCTTAT-3′; reverse, 5′-CCTGATAAGAAGTTCT CCTGGTTGTCAGTCAGTGGCC AAAACAACCAGGAGAGAACTTCTTATC-3′.

### Histological analysis

Eight weeks after the surgery, all rats were sacrificed (at 19 weeks old). The right maxillae were harvested, decalcified with 10% EDTA, embedded in paraffin, and cut into sections of 5-μm thickness for hematoxylin and eosin (H&E) staining as previously described [12]. Stained sections were photographed under a light microscope equipped with a Nikon CoolPix 4500 (Nikon Corp., Tokyo, Japan), and the level of bone tissue regeneration was analyzed according to Lane-Sandhu histological scoring criteria [13].

### Quantitative real-time reverse transcription polymerase chain reaction (qRT-PCR) analysis

Upon sacrifice, tissues from the defect of left rat maxillae were collected and were stored at − 80 °C. Total RNA of the tissues was extracted using TRIzol reagent (TaKaRa, Japan). After extraction, RNA was quantified and reverse-transcribed into cDNA with Prime-Script RT reagent Kit (TaKaRa, Japan) according to the manufacturer’s instructions [14]. The resultant cDNA products were amplified using SYBR Green qPCR Master Mix (BioTools, Jupiter, FL, USA) for real-time PCR analysis as previously reported [15]. Primer sequences used were as follows: NLRP3: 5′-ATTACCCGCCCGAGAAAGG-3′ (sense), 5′-TCGCAGCAAAGA TCCACACAG-3′ (antisense); ASC: 5′-TTGCTGGATGCTCTGTATGG-3′ (sense), 5′-CCAAGTAGGGCTGTGTTTGC-3′ (antisense); caspase-1: 5′-GGTCTTGTGACTTGG AGGACAT-3′ (sense), 5′-TTTCAGTGGTTGGCATCTGTAG-3′ (antisense); IL-1β: 5′-TGACCTGTTCTTTGAGGCTGAC-3′ (sense), 5′-GATGCTGCTGTGAGATTT GAAG-3′ (antisense); Runt-related transcription factor 2 (RUNX2): 5′-CCACCACTCA CTACCACACG-3′ (sense), 5′-TAT GGAGTGCTGCTGGTCTG-3′ (antisense); osteocalcin (OCN): 5′-CATGAGGACCCTCTCTCTGC-3′ (sense), 5′-TTCACCACCTTACTG CCCTC-3′ (antisense); and β-actin: 5′-GGCCAACCGTGAAAAGATGA-3′ (sense), 5′-GACCAGAGGCATACAGGGACAA-3′ (antisense).

### Western blot analysis

Total protein in the tissues from the defect of left rat maxillae was obtained using a ReadyPrep Protein Extraction Kit (BioRad Laboratories, Hercules, CA, USA), and inflammation-related proteins and bone formation-related proteins were detected using western blot analysis [16]. The primary antibodies were rabbit polyclonal anti-NLRP3 (1:500) and mouse monoclonal anti-glyceraldehyde 3-phosphate dehydrogenase (anti-GAPDH) (1:500), anti-ASC (1:800), anti-caspase-1 (1:800), anti-IL-1β (1:500), anti-RUNX2 (1:500), and anti-OCN (1:500). The secondary antibody was horseradish peroxidase-conjugated anti-mouse (1:2000) or anti-rabbit (1:3000). The immunoreactive bands were detected using enhanced chemiluminescence. All antibodies were from Santa Cruz Biotechnology (Santa Cruz, CA, USA), except the rabbit polyclonal primary antibody from Abcam (Cambridge, MA, USA).

### Statistical analysis

All data were digitized in Statistical Package for the Social Sciences (SPSS) software (version 23.0, SPSS Inc., Chicago, IL, USA) and were expressed as means ± SD. Statistical significance of the differences between the groups was measured by one-way analysis of variance (ANOVA) followed by the Student-Newman-Keuls *q* test. Results with *P* < 0.05 were considered statistically significant.

## Results

### Fasting blood glucose of rats

**Fig. 1:**
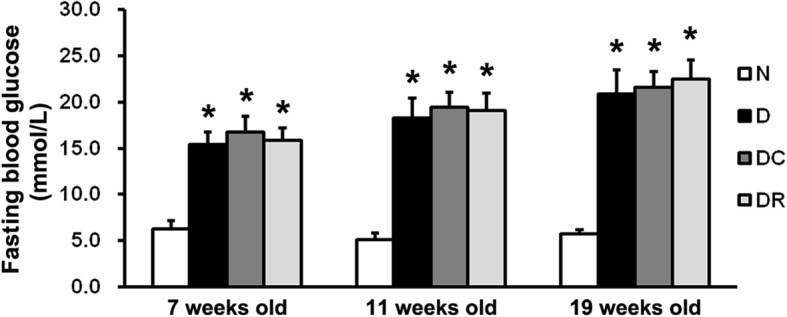
Fasting blood glucose levels of N, D, DC, and DR rats were detected at 7, 11, and 19 weeks old. Data are presented as the mean ± SD (*n* = 10, *P < 0.05 vs. N rats). N, normal control group; D, diabetes without treatment group; DC, diabetes with control shRNA lentivector treatment group; DR, diabetes with lentiviral NLRP3 shRNA treatment group

As demonstrated in Fig. 1, fasting blood glucose levels of D, DC, and DR rats were all above 13.89 mmol/L at 7 weeks old, suggesting the establishment of diabetes models. On the day of surgery and at sacrifice (at 11 and 19 weeks old), the rats in the D, DC, and DR groups also exhibited high glycemic levels, compared with normal control rats. No significant difference in fasting blood glucose level was observed among the D, DC, and DR groups at 7, 11, and 19 weeks old.

### Histological observations of alveolar bone healing after NLRP3 RNAi

**Fig. 2:**
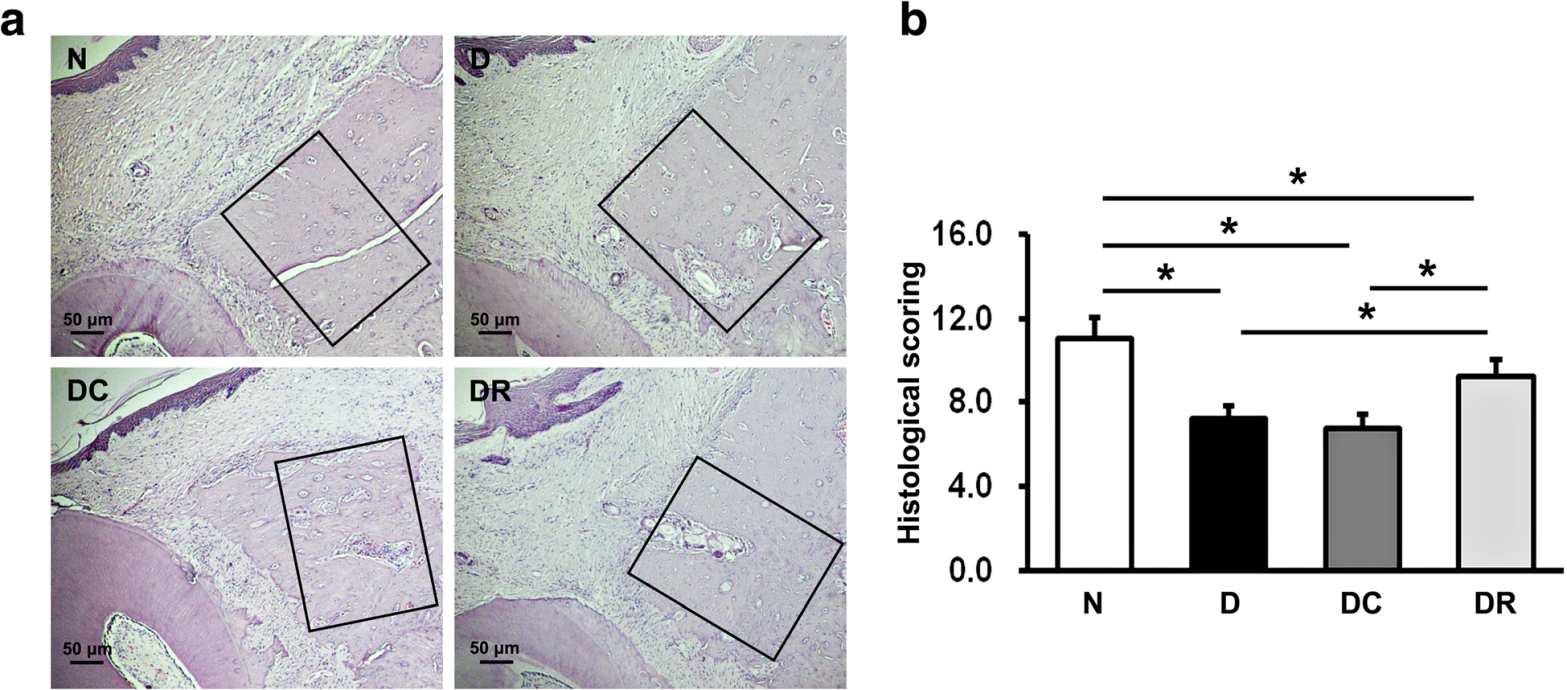
Alveolar bone defect repair of rats was examined 8 weeks after surgery using H&E staining. **a** Images of the alveolar bone defect area of N, D, DC, and DR rats. **b** Lane-Sandhu scoring of bone regeneration of N, D, DC, and DR rats (*n* = 10, *P < 0.05). N, normal control group; D, diabetes without treatment group; DC, diabetes with control shRNA lentivector treatment group; DR, diabetes with lentiviral NLRP3 shRNA treatment group

The histological sections showed more new bone formation in the defect area in N rats than in D, DC, and DR rats after 8 weeks of healing, demonstrating impaired alveolar bone defect healing under the diabetic condition. Moreover, new bone formation was greater in DR rats, compared with D and DC rats, while no visible differences were found between D and DC rats, suggesting the improvement of bone repair by NLRP3 shRNA treatment (Fig. 2a). Lane-Sandhu scoring of bone regeneration also supported these observations. The score was higher in N rats than in all diabetic rats and higher in DR rats than in D and DC rats, with no obvious difference between D and DC rats (Fig. 2b).

### Effects of NLRP3 RNAi on NLRP3 inflammasome and IL-1β expression

**Fig. 3:**
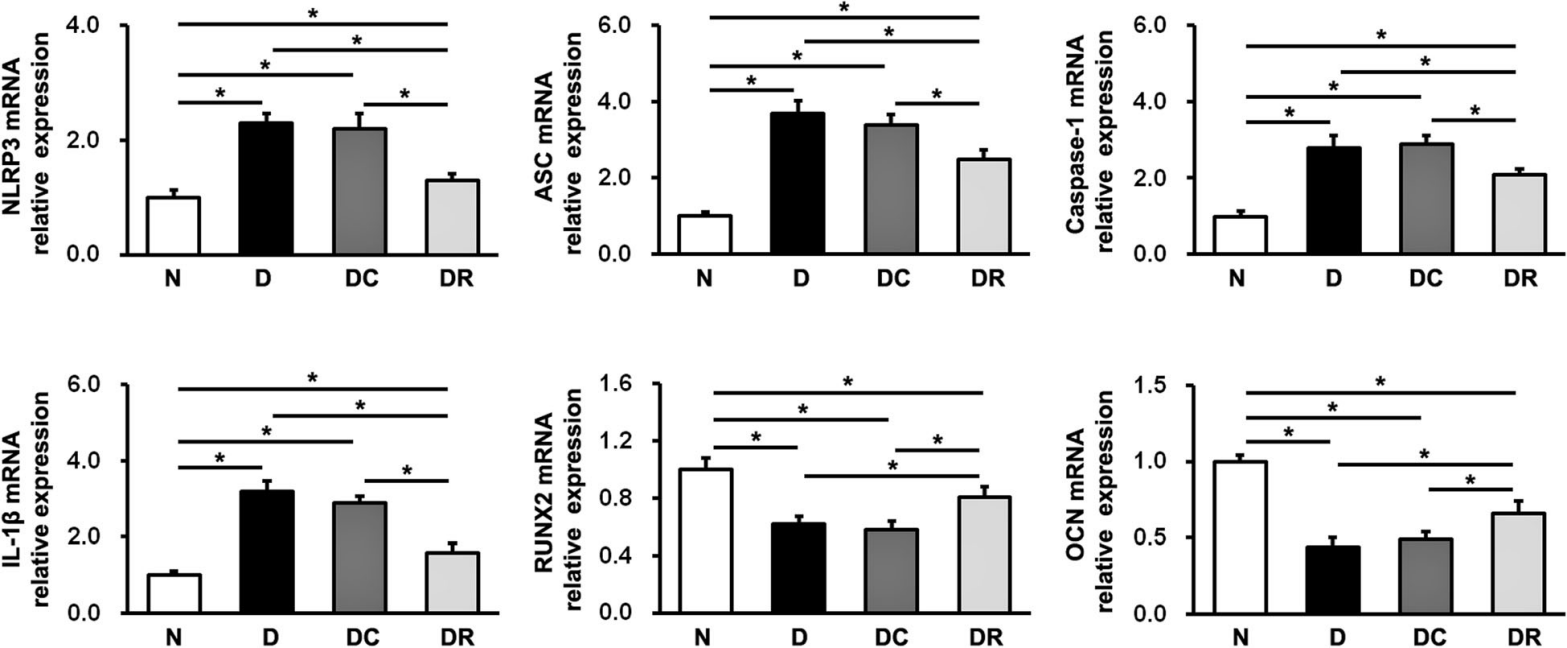
MRNA expression levels of NLRP3, ASC, caspase-1, IL-1β, RUNX2, and OCN in the alveolar bone defect area were measured using qRT-PCR analysis. Data are presented as the mean ± SD (*n* = 3, *P < 0.05). N, normal control group; D, diabetes without treatment group; DC, diabetes with control shRNA lentivector treatment group; DR, diabetes with lentiviral NLRP3 shRNA treatment group

**Fig. 4:**
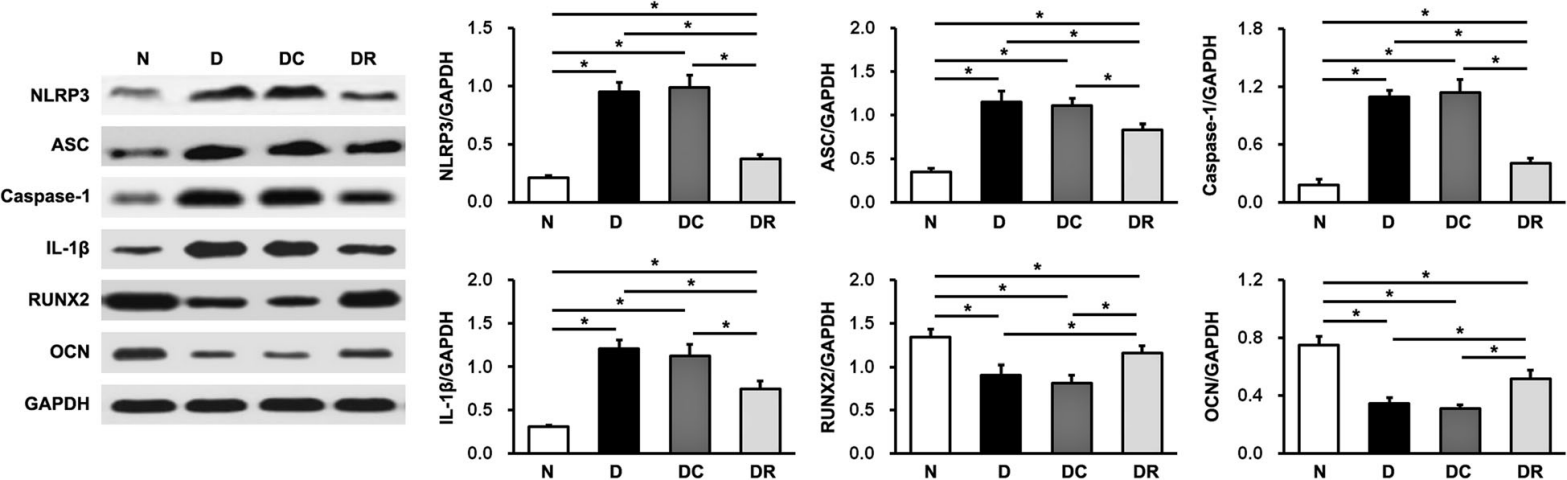
Protein expression levels of NLRP3, ASC, caspase-1, IL-1β, RUNX2, and OCN in the alveolar bone defect area were detected using western blot analysis. Data are presented as the mean ± SD (*n* = 3, *P < 0.05). N, normal control group; D, diabetes without treatment group; DC, diabetes with control shRNA lentivector treatment group; DR, diabetes with lentiviral NLRP3 shRNA treatment group

The results of qRT-PCR and western blot analyses are presented in Figs. 3 and 4. The finding of western blot analysis was consistent with that of qRT-PCR analysis. At sacrifice, the expression levels of NLRP3, ASC, and caspase-1 in the DR group were significantly lower than those in the D and DC groups, although the expression levels were higher in all diabetic groups (D, DC, and DR groups) than in the normal control group. No significant differences in the expression levels of NLRP3, ASC, or caspase-1 were detected between the D and DC groups. Additionally, the expression levels of IL-1β were in agreement with those of NLRP3, ASC, and caspase-1. IL-1β mRNA and protein expression was upregulated in all diabetic rats (D, DC, and DR rats), compared with their normal counterparts, whereas it was downregulated in DR rats, compared with D and DC rats. No differences in mRNA or protein expression of IL-1β were found between D and DC rats. These observations implicate greater NLRP3 inflammasome activation and IL-1β production in alveolar bone defect under diabetic conditions, which could be inhibited by the administration of shRNAs targeting NLRP3.

### Effects of NLRP3 RNAi on bone formation-related factor expression

RUNX2 and OCN have been considered as two important osteogenic marker genes, so we detected their expression in the alveolar bone defect area of rats using qRT-PCR and western blot analyses. As shown in Figs. 3 and 4, at 19 weeks old, both mRNA and protein expression levels of RUNX2 and OCN were decreased in D, DC, and DR rats, compared with N rats. However, the mRNA and protein expression of these two markers was enhanced in DR rats, compared with D and DC rats. There were no significant differences in RUNX2 or OCN expression between D and DC rats.

## Discussion

The mechanisms of diabetes-related impaired wound healing in soft tissues have been extensively explored [7, 17, 18], but much remains unknown on delayed alveolar bone healing under diabetic conditions. In this work, we observed that diabetic rats showed a significantly impaired alveolar bone defect healing, compared with their normal controls. Additionally, the expression of NLRP3 inflammasome was enhanced in the defect sites of diabetic rats. After the treatment of lentiviral shRNA targeting NLRP3, the activation of NLRP3 inflammasome was reduced, and the bone repair was improved. These results suggest that the delayed healing of alveolar bone in diabetes was closely correlated with elevated activation of NLRP3 inflammasome in the defect sites. An experiment on diabetic mice has demonstrated upregulation of proteins in the NLRP3 inflammasome in skin wound tissues and accelerated healing after decreasing the activity of the inflammasome [7]. Another study on diabetic animals has also shown increased NLRP3 inflammasome activity in tooth extraction sockets, which is associated with delayed wound healing around sockets [19]. Upon blocking the NLRP3 inflammasome by Ac-YVAD-cmk, a specific caspase-1 inhibitor, impeded mucosal healing with bone necrosis around the extraction sockets can be markedly improved [19].

Increasing evidence has shown that the activation of NLRP3 inflammasome cascade plays a crucial role in sustained inflammation in different inflammatory diseases, including atherosclerosis, chronic obstructive pulmonary disease, and diabetes [20–22]. NLRP3 activation can cause pro-caspase-1 cleavage and mature IL-1β release [6, 7]. Subsequently, NLRP3-dependent IL-1β secretion induces immune cells, such as macrophages, to become more proinflammatory and to release a greater amount of proinflammatory cytokines like tumor necrosis factor-α and interleukin-6 [23, 24]. Then, these proinflammatory cytokines amplify inflammatory responses in the diseased site, resulting in exacerbated tissue damage or impaired tissue regeneration [25]. In the current study, we also demonstrated the high expression level of IL-1β in the alveolar bone defect in diabetic mice, in comparison with normal control mice. Upon NLRP3 shRNA treatment, the reduced expression of IL-1β was observed accompanied with improved healing. These findings implicate a key role of NLRP3-induced IL-1β production in diabetes-associated impairment of alveolar bone defect healing. Several studies on bone diseases have shown the adverse effects of IL-1β production induced by NLRP3 inflammasome activation on the bone. NLRP3 inflammasome-caused IL-1β expression has been found positively associated with the development of bisphosphonate-related osteonecrosis in diabetes [19]. High expression levels of NLRP3 inflammasome and IL-1β deteriorate osteoporosis under estrogen deficiency by inhibiting osteogenic differentiation [26]. Moreover, the suppression of IL-1β expression by blockade of NLRP3 inflammasome activity can repress the progression of these two bone diseases [19, 26]. In periodontal diseases, upregulation of IL-1β protein and NLRP3 inflammasome has also been demonstrated in periodontal tissues of uncontrolled type 2 diabetic patients with periodontitis, who exhibit more alveolar bone damage, compared with nondiabetic periodontitis patients [8].

Bone repair is a process involving proliferation and differentiation of different osteogenic cells [27]. Several biological factors, such as RUNX2 and OCN, are expressed during the process of bone healing, and the alteration of their expression levels can affect the progression of bone restoration [28]. In this work, we observed that the expression levels of RUNX2 and OCN were reduced in the alveolar bone defect in diabetic mice, compared with the normal controls. However, the expression levels of these factors in diabetic mice were elevated after the treatment of shRNA targeting NLRP3. These findings further demonstrate the improvement of bone healing by the inhibition of NLRP3 inflammasome and IL-1β (Additional file 1: Figure S1). RUNX2 has been known as a critical transcription factor in osteoblast differentiation [29], and it is located upstream to many other osteogenic factors including OCN [30]. OCN is a bone-specific protein synthesized by osteoblasts and is vital in the formation of hydroxyapatite crystals and the progression of mineralization [31]. Previous studies have shown that the repression of RUNX2 and OCN expression is associated with the reduction of bone formation [32, 33]. IL-1β has been reported to stimulate proliferation of osteoblasts and production of mineralized bone matrix [34], but high concentrations of this cytokine suppress osteogenesis [35]. Additionally, IL-1β can stimulate the secretion of tumor necrosis factor-α and interleukin-6 by immunoregulatory cells and osteoblasts, subsequently inhibiting bone formation [25]. Reduced Runx2 expression and impaired osteogenic differentiation are observed in preosteoblastic cells after excessive IL-1β treatment [36]. Sustained IL-1β expression accompanied with delayed OCN production and osteoblast differentiation are also found in periodontal bone tissues around dental implants under diabetic conditions [1]. These published reports also implicate that the impaired bone formation may be correlated with the impeded osteogenic gene expression caused by IL-1β overexpression.

## Conclusions

In summary, we observed that a lentiviral shRNA targeting NLRP3 increased the bone regeneration in the alveolar bone defect of diabetic rats. It also inhibited the expression of NLRP3 inflammasome and IL-1β and enhanced the expression of RUNX2 and OCN. These findings indicated that NLRP3 inflammasome suppression could accelerate alveolar bone defect healing in diabetes, which may be associated with repressed proinflammatory cytokine production and enhanced osteogenic gene expression under hyperglycemic conditions.

## Additional file

Additional file 1: **Figure S1.** Regulation of alveolar bone defect healing by shRNA targeting NLRP3 under the diabetic condition. a Increased expression of NLRP3 leads to the enhanced expression of ASC and caspase-1, subsequently resulting in the augmented release of proinflammatory cytokine IL-1β and decreased production of osteogenic markers RUNX2 and OCN under the diabetic condition. b shRNA targeting NLRP3 reduces the mRNA level of NLRP3 and inhibits the process shown in Figure S1a. (TIF 1473 kb)

## Data Availability

The datasets used and/or analyzed during the present study are available from the corresponding author on reasonable request.

## Abbreviations

ASC: Apoptosis-associated speck-like protein containing a CARD
D: Diabetes without treatment
DC: Diabetes with control shRNA lentivector treatment
DR: Diabetes with lentiviral NLRP3 shRNA treatment
H&E: Hematoxylin and eosin
IL-1β: Interleukin-1β
N: Normal control
NLRP3: NACHT [nucleotide-binding oligomerization], LRR [leucine-rich repeat], and PYD [pyrin domain] domains-containing protein 3
OCN: Osteocalcin
qRT-PCR: Quantitative real-time reverse transcription polymerase chain reaction
RNAi: RNA interference
RUNX2: Runt-related transcription factor 2
shRNA: Short hairpin RNA
SPSS: Statistical Package for the Social Sciences
STZ: Streptozotocin
