# Supplementary figures and images for: Suppression of NLRP3 inflammasome improves alveolar bone defect healing in diabetic rats
Source: J Orthop Surg Res. 2019 May 30;14:167. doi: 10.1186/s13018-019-1215-9 (PMC6543640; doi:10.1186/s13018-019-1215-9)

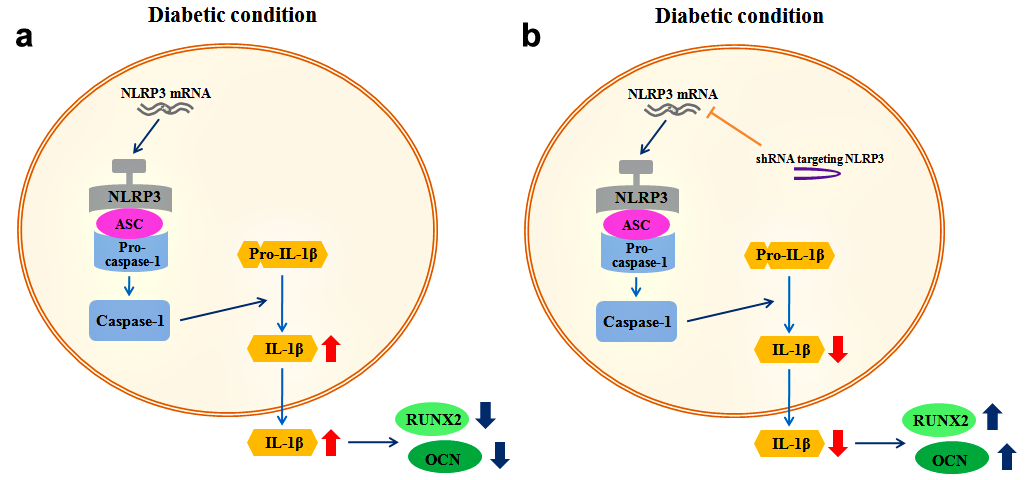

Supplement: Supplementary file 1 — Figure S1. Regulation of alveolar bone defect healing by shRNA targeting NLRP3 under the diabetic condition. a Increased expression of NLRP3 leads to the enhanced expression of ASC and caspase-1, subsequently resulting in the augmented release of proinflammatory cytokine IL-1β and decreased production of osteogenic markers RUNX2 and OCN under the diabetic condition. b shRNA targeting NLRP3 reduces the mRNA level of NLRP3 and inhibits the process shown in Figure S1a. (TIF 1473 kb) [file 13018_2019_1215_MOESM1_ESM.tif]
